# Epulo-Cancroid Tumor

**Published:** 1861-07

**Authors:** 


*July* 2d—Prof. Andrews, at his clinique at the *Mercy*, this morning, removed an epulo-cancroid tumor, from the lower jaw of a German woman, which had involved one of the submaxillary glands and the alveolar process of the four incisors—necessitating its excision.

